# Structural bias in vitamin A metabolism: Why α-retinoids miss the eye

**DOI:** 10.1016/j.jbc.2025.110713

**Published:** 2025-09-11

**Authors:** Sepalika Bandara, Aicha Saadane, Pranesh Ravichandran, Srinivasagan Ramkumar, Johannes von Lintig

**Affiliations:** 1 Department of Pharmacology, School of Medicine, Case Western Reserve University, Cleveland, Ohio, USA; 2 Department of Surgery, University of Illinois College of Medicine at Peoria, Peoria, Illinois, USA; 3 Disease Biology Laboratory, Baush & Lomb Incorporated, Irvine, California, USA

**Keywords:** metabolism, retinoids, vision, vitamin A, lipid transport

## Abstract

Provitamin A carotenoids are metabolized to retinoids, critical for vision and transcriptional regulation, through oxidative cleavage by carotenoid oxygenases. β-Carotene, a symmetric carotenoid, undergoes central cleavage by β-carotene oxygenase 1 (BCO1), generating two molecules of retinaldehyde. In contrast, the metabolism of asymmetric carotenoids, such as α-carotene (β,ε-carotene) and β-cryptoxanthin (β,β-carotene-3-ol), produces noncanonical apocarotenoid derivatives in addition to retinaldehyde. Here, we dissect the enzymatic pathways and transport mechanisms governing these metabolic fates in mice. We demonstrate that α-carotene is cleaved exclusively by BCO1 to yield retinaldehyde and α-retinaldehyde, bypassing mitochondrial processing. β-Cryptoxanthin, however, undergoes an initial eccentric cleavage by mitochondrial BCO2, followed by cytosolic BCO1-mediated central cleavage, producing only retinaldehyde. This divergence arises from differential subcellular trafficking: β-cryptoxanthin is transported to mitochondria *via* Aster-B, while α-carotene is excluded. Downstream, α-retinol is esterified by lecithin: retinol acyltransferase (LRAT), trafficked in chylomicrons, and stored as α-retinyl esters in the liver under ISX-mediated transcriptional control. Notably, α-retinol is not mobilized into circulation *via* retinol-binding protein 4 (RBP4), and genetic ablation of its receptor, STRA6, does not alter α-retinyl ester storage in lung tissue. Intriguingly, α-retinyl esters accumulate in the eyes of STRA6-deficient mice yet fail to participate in the visual cycle due to exclusion from RPE65-mediated isomerization. These findings establish α-retinoids as metabolic tracers of BCO1 activity and chylomicron-mediated vitamin A delivery and reveal mechanistic safeguards that prevent incorporation of noncanonical retinoids into the visual cycle.

Vitamin A is an essential nutrient that supports vision, immunity, reproduction, and cellular homeostasis (1, 2, 3, 4). It is obtained from preformed retinoids in animal products or from provitamin A carotenoids found in plant-based foods. With the growing popularity of vegetarian and vegan diets, provitamin A carotenoids particularly β-carotene, β-cryptoxanthin, and α-carotene have become increasingly important sources of dietary vitamin A (5, 6). These carotenoids are enzymatically cleaved to produce retinaldehyde (7), a central intermediate in retinoid metabolism and the precursor for both visual chromophore and retinoic acid.

The metabolism of carotenoids is determined by the subcellular localization of two carotenoid cleavage enzymes (8). β-Carotene-oxygenase 1 (BCO1) resides in the cytosol and accesses cytoplasmic membrane-associated substrates (9), while β-carotene-oxygenase 2 (BCO2) localizes to mitochondria (10). Following cleavage in enterocytes, retinoids are esterified by lecithin: retinol acyltransferase (LRAT) and incorporated into chylomicrons for systemic distribution (11, 12). In contrast, postprandial vitamin A delivery relies on hepatic stores, which are mobilized as retinol bound to RBP4 and delivered to target tissues *via* STRA6-mediated uptake (13, 14).

Among provitamin A carotenoids, α-carotene occupies a unique position as it not only serves as a source of vitamin A but also gives rise to noncanonical α-retinoids, a class of metabolites with largely unknown physiological roles. Previous studies in animal models, such as piglets and gerbils, have shown that α-retinoids accumulate in tissues as retinyl esters (REs) but remain undetectable in circulation (15, 16). This metabolic signature has been interpreted as evidence for chylomicron-mediated delivery of dietary vitamin A to peripheral tissues, bypassing hepatic mobilization *via* retinol-binding protein 4 (RBP4) (17, 18). However, these findings have not been systematically investigated in mice, a model system that enables genetic manipulation and detailed mechanistic analyses.

Therefore, the enzymatic pathways underlying α-carotene metabolism remain incompletely defined. In contrast to β-carotene, which undergoes symmetric cleavage by β-carotene oxygenase 1 (BCO1) (17, 19), and β-cryptoxanthin, which is sequentially cleaved by mitochondrial BCO2 and cytosolic BCO1 (20), it is still uncertain whether α-carotene requires both enzymes or is exclusively processed by BCO1. This gap in knowledge limits our understanding of how α-retinoids are formed and regulated.

The intestine-specific transcription factor ISX provides an additional layer of metabolic control by repressing the expression of SR-B1, a carotene uptake transporter (21, 22), and BCO1 in response to increasing systemic retinoid levels (23, 24). While ISX has been established as a key regulator of intestinal β-carotene metabolism, its role in modulating α-carotene uptake and cleavage remains unexplored.

Importantly, it is not yet clear whether α-retinoids can affect the retina, the tissue with the highest retinoid demand in the body (25). Understanding whether α-carotene-derived metabolites contribute to ocular retinoid pools or are actively excluded from them may shed light on tissue-specific vitamin A handling and reveal novel regulatory mechanisms that protect the visual cycle from noncanonical retinoid interference.

In this study, we systematically dissect the enzymatic conversion, subcellular trafficking, and tissue distribution of α-carotene–derived retinoids in mice. We clarify the specific roles of BCO1 and BCO2 in α-carotene cleavage, examine the transport protein dependencies of this pathway, and evaluate how ISX and STRA6 influence α-retinoid bioavailability. These findings establish the metabolic framework of α-carotene and highlight its utility as a tracer for ISX-regulated, chylomicron-mediated vitamin A transport.

## Results

### Enzymatic processing of α-carotene by BCO1 and BCO2

**Figure 1:**
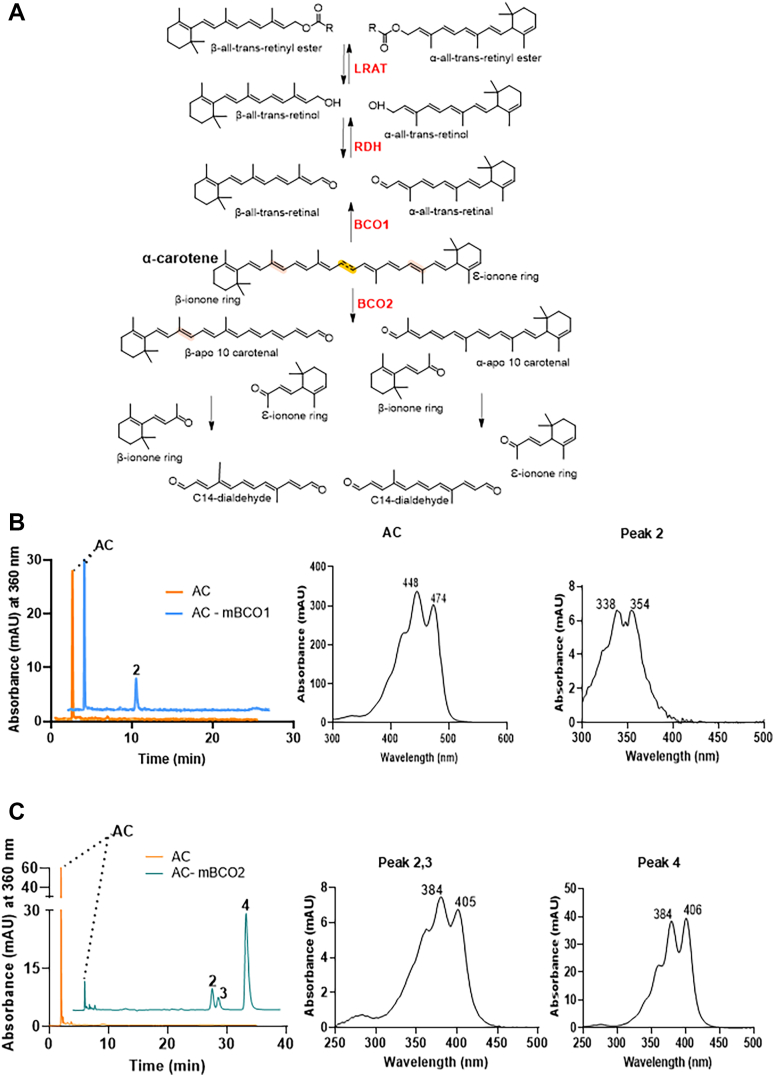
**Conversion of α-carotene by recombinant murine BCO1 and BCO2 enzymes.***A*, α-Carotene metabolites upon enzymatic conversion by BCO1, BCO2, LRAT and RDH. *B*, HPLC traces of lipophilic extracts of enzyme assays with BCO1 and α-carotene (AC) (*blue trace*) as compared to AC incubated in the absence of the BCO1 enzyme. Peak 2 represents the product peak that is a mixture of retinaldehyde-oxime and α-retinaldehyde-oxime. Spectral properties of the AC substrate and the product peak are shown on the *left*. *C*, HPLC traces of lipophilic extracts of enzyme assays with BCO2 and α-carotene (AC) (*green trace*) as compared to AC incubated in the absence of enzyme. Peak 2, 3, and 4 represent the C14-dialdehyde product peaks that are formed after the removal of the ε- and β-ionone rings of the AC substrate. Spectral properties of the AC substrate and the product peaks are shown on the *left*. HPLC system 1 was used for these analyses.

To investigate the enzymatic processing of α-carotene, we performed *in vitro* enzyme activity assays with BCO1 and BCO2. For this purpose, we expressed murine BCO1 and BCO2 as MBP-fusion proteins in *E. coli* and purified the recombinant proteins on amylose affinity columns (Fig. S1*A*). When α-carotene was used as a substrate, BCO1 should cleave it into α-retinal and retinal, producing two conformational isomers of retinaldehyde (Figs. 1, *A* and *B* and S1*B*). Analysis by HPLC system 1 revealed that the corresponding syn oximes of these two products comigrated and eluted as a single peak at approximately 10 min (Fig. 1*B*). The spectral characteristics of these retinoids were further analyzed using UV-visible spectroscopy. The absorption spectrum of the retinaldehyde oxime products exhibited two distinct peak maxima at 338 nm and 354 nm (Fig. 1*B*). This spectral profile was notably different from that of retinaldehyde alone, which has a single peak maximum at 356 nm (Fig. S2), indicating the presence of two retinal oxime isomers with different spectral properties. These findings showed that α-carotene served as a substrate for recombinant mouse BCO1, leading to the production of both α-retinaldehyde and retinaldehyde (Fig. 1*A*).

Unlike BCO1, which catalyzes central cleavage, BCO2 cleaves across C9, C10 of carotenoids, yielding a variety of metabolites (Fig. 1*A*). When α-carotene was incubated with BCO2, the putative cleavage products were apo-10′-carotenals, a C14-dialdehyde, and α- and ε-ionones. Analysis on HPLC system 1 of the reaction products revealed the presence of three distinct peaks with similar spectral properties (Fig. 1*C*). These products eluted at approximately 30 min, appearing as three separate peaks, suggesting the existence of at least three prominent isomers (Fig. 1*C*). UV-visible spectral analysis of the products revealed absorption maxima at 384 nm and 405 nm, indicating that they likely correspond to the same molecular species or closely related isomeric forms (Fig. 1*C*). Their elution time and spectral characteristics resembled those of the C14-dialdehyde product in which both ionone ring sites are removed from the substrate (Fig. 1*A*). This outcome suggested that BCO2-mediated cleavage of α-carotene produced a set of C14-dialdehyde isomers and two ionones. The latter products were not detected because of their volatile characteristics.

Taken together, our enzymatic assays highlighted the differential processing of α-carotene by BCO1 and BCO2. While BCO1 cleaves α-carotene symmetrically, yielding α-retinal and retinal, BCO2 performs eccentric cleavage, generating dialdehyde products and ionones. The presence of multiple peaks in the chromatographic analysis suggests structural isomerism among the cleavage products.

### BCO1 is the principal α-carotene cleavage enzyme in mice

**Figure 2:**
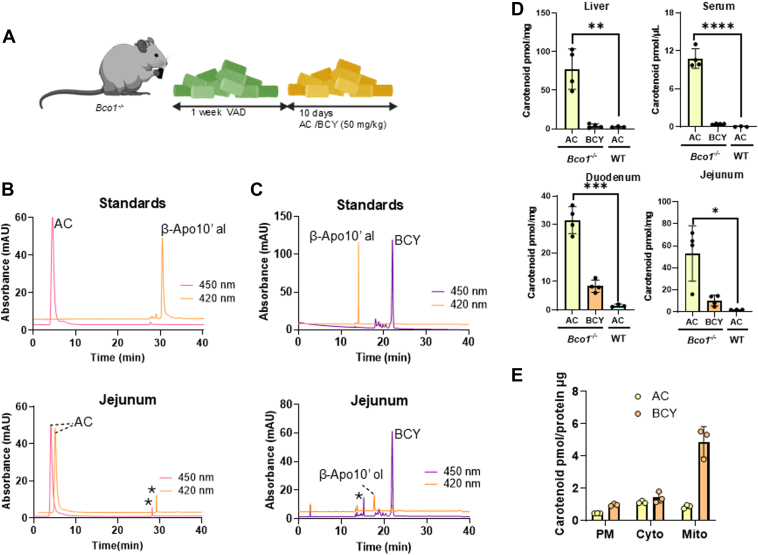
**BCO1 is the principal α-carotene metabolizing enzyme in mice.***A*, schematic representation of mouse feeding experiment. *B*, *Top panel*: HPLC traces of α-carotene (AC, *red trace*) at 450 nm and β-apo-10′-carotenal (Apo10al, *orange trace*) at 420 nm. *Lower panel*, HPLC traces of jejunum extracts of *Bco1*^*−/−*^ mice fed with AC. Separation was achieved on HPLC system 2. *C*, *top panel*: HPLC traces of β-cryptoxanthin (BCY, *purple trace*) at 450 nm and β-apo-10′-carotenal (Apo10′ al, *orange trace*) at 420 nm. *Lower panel*, HPLC traces of jejunum. extracts of *Bco1*^*−/−*^ mice fed with BCY. The *asterisk* in the HPLC traces indicate the shift in the solvent of the gradient HPLC system. Separation was achieved on HPLC system 3. *D*, comparison of AC and BCY concentrations in select tissues of *Bco1*^*−/−*^ mice compared to wild type (WT) mice after carotenoid supplementation (n = 4 per feeding condition). *E*, carotenoid concentrations in subcellular fractions of A549 cells after uptake assays for AC and BCY, respectively. (The results represent 3 independent assays per carotenoid). PM, plasma membrane, Cyto, cytoplasm, and Mito, mitochondrial fraction. In (*D* and *E*), separation of compounds was achieved on HPLC system 2 and 3, respectively. The individual data points in bar graphs represent biological replicates.

To determine which carotenoid cleavage dioxygenase (CCD) is responsible for α-carotene metabolism *in vivo*, we utilized BCO1-deficient (*Bco1*^*−/−*^) mice (17) and wild-type control mice on the same genetic background. *Bco1*^*−/−*^ mice lack functional BCO1 (17), allowing us to assess whether BCO2 compensates for α-carotene processing in this mutant. To investigate the metabolism of α-carotene, we subjected mice to a 1-week washout phase on a rodent growth diet devoid of vitamin A, ensuring depletion of pre-existing dietary carotenoids and retinoids. Following this, we fed the mice the same diet supplemented with α-carotene (50 mg/kg) for 10 days (Fig. 2*A*). Additionally, a separate cohort of *Bco1*^*−/−*^ mice was supplemented with β-cryptoxanthin at an equivalent dose following the same intervention scheme. After the dietary intervention, we analyzed carotenoid concentrations and their metabolites in tissues and blood.

This analysis revealed that α-carotene accumulated in the jejunum, blood, and liver of *Bco1*^*−/−*^ mice (Fig. 2, *B* and *D*). In contrast, no such accumulation of α-carotene was observed in WT mice. Instead, we detected significant amounts of α-retinoids in the liver and lungs of WT animals. To detect these compounds, we established an HPLC system with a chiral column and a stepwise gradient that separated α-REs and α-retinol from canonical retinoids based on retention times (Fig. S1, *A* and *B*).

The findings in *Bco1*^*−/−*^ mice suggested that BCO2 does not effectively metabolize α-carotene, allowing the provitamin to accumulate rather than undergo metabolic conversion. These findings contrast with previous findings with β-cryptoxanthin, which is metabolized to retinaldehyde by stepwise cleavage by BCO1 and BCO2 (20). In fact, β-cryptoxanthin did not accumulate in *Bco1*^*−/−*^ mice despite being supplemented at the same level as α-carotene (Fig. 2, *C* and *D*). Instead, analysis of lipid extracts from the jejunum revealed that β-10′-apocarotenol was present (Fig. 2*C*). This long-chain apocarotenoid results from BCO2-mediated cleavage at the C9′, C10′ double bond adjacent to the 3-hydroxy-ionone ring of β-cryptoxanthin. The accumulation of this specific cleavage product indicated that BCO2 efficiently processes β-cryptoxanthin, preventing its accumulation in tissues (Fig. 2*D*).

Together, the analyses in mice highlighted distinct metabolic pathways for asymmetric provitamin A carotenoids α-carotene and β-cryptoxanthin. While BCO1 is essential for α-carotene metabolism, BCO2 alone does not cleave α-carotene into products, resulting in its accumulation in mouse tissues. In contrast, β-cryptoxanthin is converted by BCO2 to β-10′-apocarotenoids, preventing its accumulation in the mouse tissues deficient for BCO1.

### Subcellular transport of α-carotene and β-cryptoxanthin

To investigate the biochemical basis of the differing metabolic fates of α-carotene and β-cryptoxanthin, we examined whether their intracellular transport is influenced by Aster proteins. Previous studies have shown that the metabolism of xanthophylls, but not carotenes, is modulated by these transport proteins in murine enterocytes (26). Given that β-cryptoxanthin is a xanthophyll while α-carotene is a carotene, we hypothesized that their distinct metabolic handling, respectively by BCO1 and BCO2, might be linked to differences in transport mechanisms.

To explore this, we studied the intracellular trafficking of α-carotene and β-cryptoxanthin in A549 cells, a human lung carcinoma cell line that expresses the *GRAMD1B* gene, which encodes Aster-B (27). We employed an established assay to monitor the transport of carotenoids from the plasma membrane to mitochondria, a process relevant to BCO2 activity (8). Following incubation with either α-carotene or β-cryptoxanthin, we fractioned the cells into plasma membrane, cytoplasm, and mitochondria to determine the intracellular distribution of each carotenoid.

Our results showed that β-cryptoxanthin was abundantly present in the mitochondria, with only minor amounts detected in the plasma membrane and cytoplasm (Fig. 2*E*). Notably, α-carotene was not accumulating in mitochondria, suggesting limited access to BCO2-mediated cleavage despite the enzyme’s demonstrated ability to process α-carotene *in vitro* (Figs. 1*D* and 2*E*). These findings suggested that Aster proteins play a crucial role in β-cryptoxanthin transport to mitochondria, enabling its subsequent metabolism by BCO2. In contrast, α-carotene remains in the cytoplasm and is not efficiently trafficked to the organelles, explaining why it accumulates in *Bco1*^*−/−*^ mice despite being a substrate for BCO2 in enzymatic assays.

### ISX is a key controller of α-carotene metabolism

**Figure 3:**
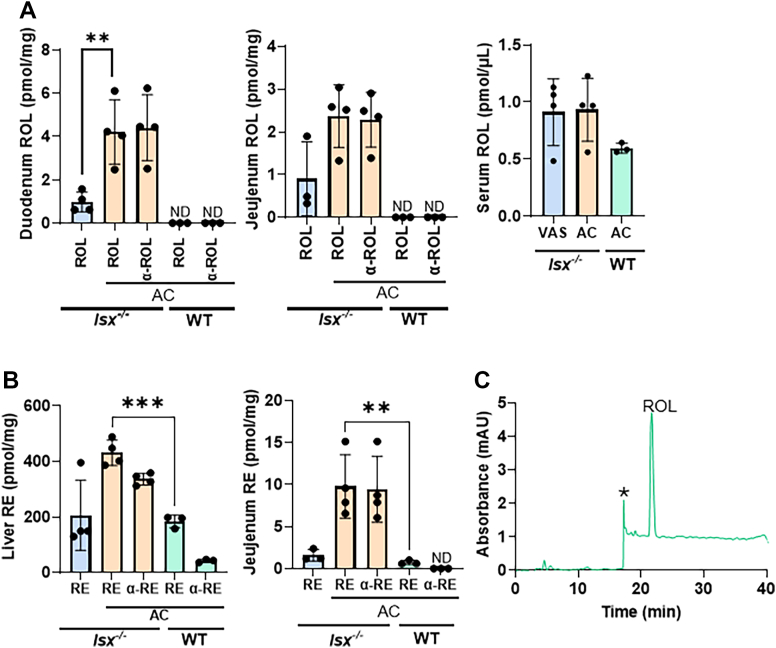
**α-Carotene metabolism by *Isx*^*−/−*^ mice.***A* and *B*, *Isx*^*−/−*^ mice were fed with vitamin A sufficient diet (*blue bars*) or with α-carotene diet (AC, *orange bars*) and wild type(WT) mice fed with α-carotene diet (AC, *green bars*). Amounts of retinoids (ROL, α-ROL, RE, and α-RE) were determined by chiral column HPLC (n = 4 animals per tissue and feeding condition). *C*, HPLC chromatogram at 325 nm of serum extracts of AC fed *Isx*^*−/−*^ mice revealed the presence of ROL but not α-ROL. Separation was achieved on HPLC system 4. The individual data points in bar graphs represent biological replicates.

To further explore α-carotene metabolism, we next examined mice that lack intestine-specific homeobox (*Isx*^*−/−*^ mice), a transcription factor that suppresses SR-B1 and BCO1 expression in intestinal enterocytes (23, 24, 28). To test whether ISX-deficiency enhances α-carotene conversion, we subjected *Isx*^*−/−*^ and WT mice to the same dietary intervention protocol as *Bco1*^*−/−*^ mice (Fig. 2*A*). Additionally, to compare the metabolic handling of provitamin A carotenoids and preformed vitamin A, a separate group of mice received the same diet with vitamin A supplementation (VAS) (4000 IU/kg). After 2 weeks, *Isx*^*−/−*^ mice exhibited a significant increase in total retinoids (α-retinoids and retinoids) in the jejunum and liver when compared to WT mice fed the same diet (Fig. 3, *A* and *B*), indicating that ISX-deficiency significantly influenced the absorption and conversion of α-carotene. Moreover, intestinal and hepatic retinoid levels were significantly higher in *Isx*^*−/−*^ mice supplemented with α-carotene than in the same mice supplemented with preformed vitamin A (Fig. 3, *A* and *B*). We again detected no α-retinol in the serum of α-carotene-supplemented *Isx*^*−/−*^ and WT mice (Fig. 3, *A* and *C*), suggesting that it is not transported by RBP4.

These findings indicate that α-carotene bioavailability is regulated by ISX. In ISX-deficient mice, enhanced intestinal uptake leads to substantial conversion of α-carotene, as reflected by marked increases in α-retinyl esters in the liver as compared to WT mice. Notably, α-retinol remained undetectable in the blood, confirming that it is not transported by RBP4, even under conditions of elevated dietary supply and increased metabolic conversion. This lack of binding to RBP4 has been originally reported for the purified protein and later confirmed in animal models (15, 29).

### LRAT catalyzes the esterification of α-retinol

**Figure 4:**
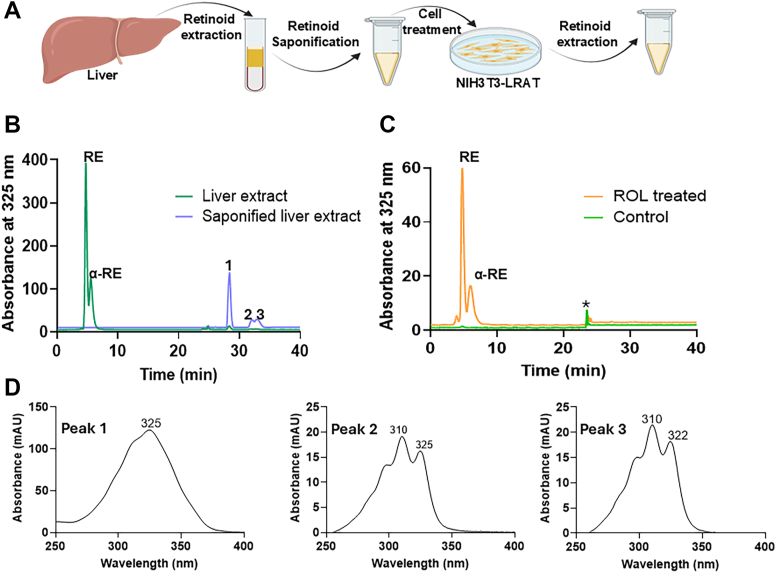
**LRAT converts α-retinol into α-retinyl esters.***A*, Schematic representation of the different steps of retinol/α-retinol assay in NIH-3T3- LRAT cells. *B*, HPLC chromatograms of extracted liver retinoids of mice fed with α-carotene before (*green*) and after saponification (*blue*). *C*, incubation of NIH3T3-LRAT cells with hepatic retinol/α-retinol extracts results in the formation of retinyl esters (REs) and α-retinyl esters (α-REs). *D*, UV-visible spectra of the ROL and α-ROL peaks of the saponified liver retinoids. The *asterisks* (∗) mark the change in solvent composition during gradient elution on the HPLC system. Separation was achieved on HPLC system 4.

LRAT is the major enzyme for RE formation for chylomicron transport as well as pulmonary and hepatic storage (11, 30). To test whether LRAT catalyzed α-RE formation, we took advantage of a previously established NIH3T3 cell stably expressing human LRAT (31). Because we lacked a commercial source for α-retinol, we extracted α-RE from mouse liver and subjected the liver extracts to saponification to hydrolyze REs into free α-retinol and retinol (Fig. 4*A*). HPLC analysis of the liver extract revealed a predominant presence of retinyl esters, while the saponified sample contained both retinol and α-retinol (Fig. 4, *B* and *D*). This retinol mixture was subsequently used to treat NIH3T3 cells expressing LRAT to assess the enzyme’s catalytic activity toward these compounds. Analysis of the treated cells demonstrated the formation of retinyl esters, with both REs and α-REs detected in HPLC analysis (Fig. 4*C*). These findings indicated that LRAT is capable of esterifying both retinol and α-retinol, suggesting that LRAT does not exhibit strong substrate selectivity for either retinoid. Thus, we provide evidence that LRAT contributes to α-carotene metabolism, particularly to the production of α-RE in tissues.

### Analysis in STRA6-deficient mice demonstrates RBP4-independent distribution of α-retinol

**Figure 5:**
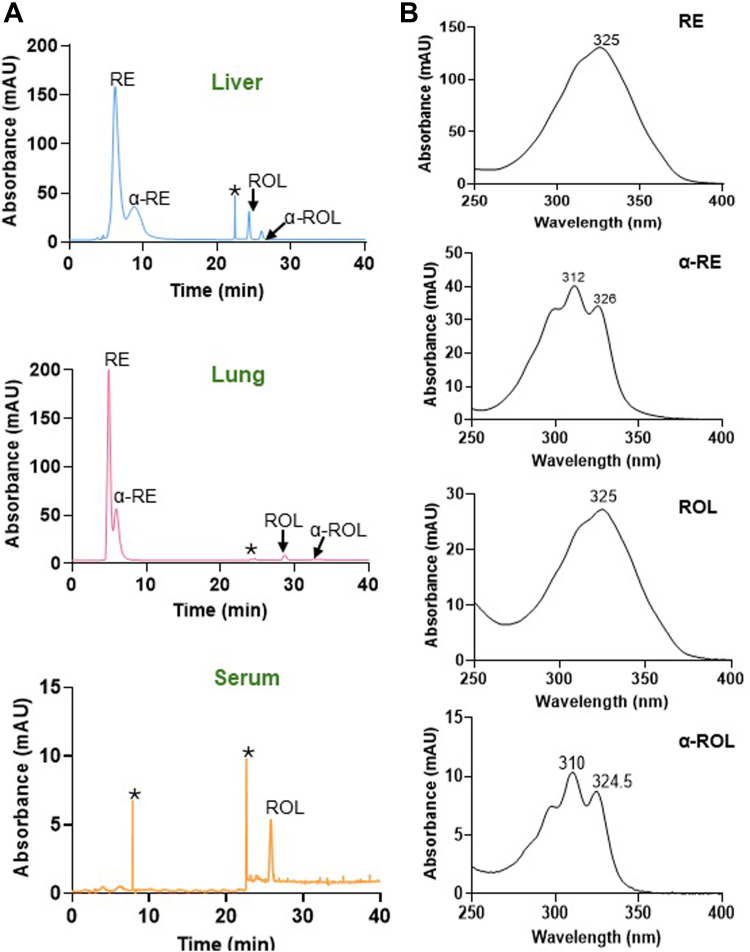
**α-Carotene metabolism of *Isx*^*−/−*^/*Stra6*^*−/−*^ mice.***A*, HPLC chromatogram of tissue extracts of *Isx*^*−/−*^/*Stra6*^*−/−*^ mice fed with α-carotene. The retentions times of α-retinol (α-ROL) and α-retinyl esters (α-REs) and retinol (ROL) and retinyl esters (RE) is indicated. The *asterisks* (∗) mark a peak that results from the change in solvent composition during the gradient elution. *B*, UV-visible spectra of RE, α-RE, ROL and α-ROL. Separation was achieved on HPLC system 4.

We observed no α-retinol in the blood of the mice, suggesting that α-retinoids are distributed *via* the chylomicron pathway as previously suggested in piglets receiving α-retinol supplementation (4, 15). To demonstrate that α-retinol delivery to peripheral tissues is independent of RBP4, we exploited *Stra6*^−/−/^*Isx*^*−/−*^ double knockout (DKO) mice. These mice lack STRA6, the RBP4 receptor, and show enhanced chylomicron-mediated delivery of dietary retinoids to the periphery (32). Upon α-carotene feeding using our standard protocol, we measured α-retinoid levels across tissues in the mice. HPLC analysis revealed high levels of α-retinol and α-RE in the liver of DKO mice (Fig. 5, *A* and *B*). α-Retinol and α-RE were also detectable in the lung (Fig. 5, *A* and *B*), confirming that these retinoids reached peripheral tissues independently of the RBP4 and STRA6-mediated pathway.

### Ocular metabolism of α-retinoids

**Figure 6:**
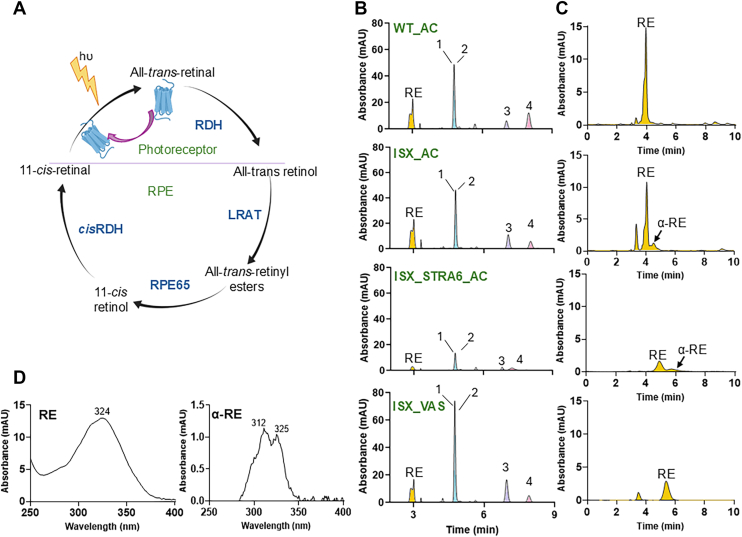
**Ocular retinoid composition of different mouse strain fed with α-Carotene.** Mice were subjected to feeding with α-carotene as described in Figure 2*A*. Upon sacrifice, eyes were enucleated. Retinoids were extracted and subjected to HPLC analysis. *A*, scheme of the intermediates of the visual cycle. *B*, HPLC chromatograms of extracts of ocular retinoids extracts separated with HPLC system 5. Peak 1: all-*trans*-retinal oxime (syn), peak 2: 11-*cis*-retinal oxime (syn), peak 3: 11-*cis*-retinal oxime (anti), and peak 4: all-*trans*-retinal oxime (anti). *C*, HPLC chromatograms of extracts of ocular retinoids extracts separated with HPLC system 4. *D*, UV-visible spectra of RE and α-RE in the HPLC chromatograms of (C).

The eye’s high demand for vitamin A is sustained by overlapping transport pathways (33). The primary route involves RBP4–STRA6-mediated delivery through the retinal pigment epithelium (RPE), while chylomicron-mediated uptake can partially compensate if this pathway fails in RBP4 and STRA6-deficiency (13, 14, 34). Once in the RPE, retinol is esterified by LRAT, and the resulting retinyl esters (REs) are substrates for the isomerase RPE65, producing 11-*cis*-configured retinoids that are essential for vision (Fig. 6*A*) (35, 36).

To assess the contribution of these pathways to ocular retinoid homeostasis in the context of α-carotene metabolism, we analyzed retinoid profiles in different mouse lines using two chiral HPLC systems for the separation of REs and retinaldehydes. As control, we used WT mice that were supplemented with an α-carotene-supplemented diet.

In WT mice, ocular retinoids consisted of 11-*cis*- and all-*trans*-retinal along with one form of RE. Notably, α-retinoids such as α-retinaldehyde and α-RE were undetectable in ocular lipid extracts (Fig. 6*B*).

In ISX-deficient mice, we again detected 11-*cis*- and all-*trans*-retinal as major retinoid cycle intermediates. However, in contrast to WT mice, some α-REs were present in the eyes of these mice, indicating that chylomicron-derived α-retinoids can access ocular tissue in this mouse mutant (Fig. 6*C*).

In DKO mice, which rely exclusively on chylomicron-mediated retinoid delivery (37), overall ocular retinoid levels were lower than in the other mice. Interestingly, 11-*cis*- and all-*trans*-retinal remained the predominant visual cycle intermediates, though low amounts of α-RE were detected in the ocular lipid extracts (Fig. 6*D*). Importantly, α-retinaldehyde and other α-retinoid derivatives were absent, suggesting selective exclusion or limited processing of α-REs in the eyes of STRA6-deficient mice.

### Structural features may exclude ε-ionone ring interaction with CCDs

The absence of α-retinoids in the eyes of WT mice suggested that the eyes receive their retinoids mainly from circulating RBP4. This mechanism may safeguard the eyes from noncanonical retinoids, since RBP4 excludes these compounds from transport. In fact, phototransduction relies on the precise recycling of all-*trans* and 11-*cis*-retinal, and α-retinoids may interfere with this process, potentially impairing vision.

In *Isx*^*−/−*^ and DKO mice, with dysregulated retinoid delivery *via* chylomicrons, α-RE levels rose in peripheral tissues but remained low in the eye. Importantly, no α-retinaldehydes became detectable in the eyes of these mice. This finding indicated that the eye employs an additional mechanism to prevent α-retinoid processing, independent of RBP4 exclusion. A possible explanation includes that α-ionone ring is converted to an β-ionone ring during the RPE65-mediated isomerization reaction. A chemical comparable RPE65-mediated catalysis has been proposed for the conversion of lutein (β,ε-carotene-3R,3′R-diol) to meso-zeaxanthin (β,β-carotene-3R,3′S-diol) (38). To test whether RPE65 may catalyze such a ring modification, we performed experiments with NIH3T3 cells engineered to express LRAT alone or LRAT together with RPE65 (39). Retinoids were provided from liver extracts of *Isx*^*−/−*^ mice fed with α-carotene. In cells expressing only LRAT, retinoids are expected to be esterified. By contrast, if RPE65 could convert α-retinol into retinol, co-expression of LRAT and RPE65 should shift the ratio of α-retinyl esters to retinyl esters in favor of retinyl esters. However, under the conditions tested, we observed no such shift; the ratios remained unchanged, indicating that RPE65 does not catalyze this conversion (Fig. S5).

**Figure 7:**
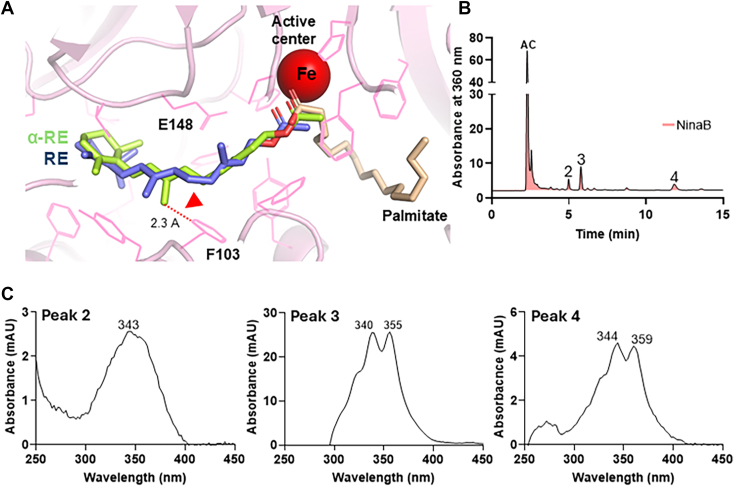
**Evidence that Carotenoid Cleavage oxygenases interact with the β-ionone ring sites of their substrates.** A, retinyl acetate (RE, *blue*) and α-retinyl acetate (α-RE, *green*) docked into the substrate binding cavity of RPE65. The arrow indicates a predicted steric clash between α-RE and amino acid side chain in the substrate tunnel. B, HPLC chromatogram at 360 nm of extracts of enzyme assays with recombinant NinaB enzyme and α-carotene (AC) separated with HPLC system 1. C, UV/Vis spectra of AC cleavage products, peak 1, 11-*cis*-retinal-oxime, peak 2, all-*trans*-α-retinal oxime (syn) and peak 3, all-*trans*-α-retinal oxime (anti).

Additionally, molecular docking studies revealed that α-REs are sterically hindered in the RPE65 binding site, and were predicted to collide with key residues (F103 and E148) for the isomerization reaction in the substrate tunnel of the enzyme (Fig. 7*A*) (40).

We also tested how insect NinaB (41, 42), an RPE65-related enzyme, processes α-carotene. For this purpose, we expressed recombinant NinaB. Interestingly, NinaB converted α-carotene into 11-*cis*-retinal and all-*trans*-α-retinal (detected as syn/anti oximes) but notably did not generate α-11-*cis*-retinal from the carotene precursor molecule (Fig. 7, *B* and *C*). These results suggested that the ε-ionone ring site, with an interrupted polyene chromophore, cannot undergo *cis*–to-*trans* isomerization when metabolized by NinaB. This finding is consistent with previous studies showing that shortening of the polyene chromophore in 5,6-dihydro-retinol prevents the isomerization of the corresponding RE (43).

These findings imply that ε-ionone ring–containing retinoids like α-retinol are excluded from the visual cycle by substrate discrimination of RPE65 in vertebrates and NinaB in arthropods.

## Discussion

Our study provides insights into metabolism, intracellular trafficking, and tissue distribution of asymmetric provitamin A carotenoids, revealing distinct enzymatic pathways and transport mechanisms that determine their physiological fate. We demonstrate that β-cryptoxanthin undergoes sequential cleavage by mitochondrial BCO2 and cytosolic BCO1 to produce canonical retinoids (19, 20), whereas α-carotene metabolism is restricted to BCO1 in the cytosol and yields both retinaldehyde and noncanonical α-retinoids. This divergence arises not from substrate specificity of the two distinct carotenoid oxygenases, since both enzymes efficiently converted α-carotene in the test tube. It is rather caused by an interplay between enzyme localization and intracellular transport by Aster proteins. Our experiments in A549 cells highlight the role of Aster-B in directing β-cryptoxanthin to mitochondria, enabling BCO2-mediated cleavage. In contrast, α-carotene remained cytosolic due to the absence of an equivalent transport mechanism, precluding access to mitochondrial BCO2 despite being a substrate *in vitro*. Thus, subcellular compartmentalization and selective transport, rather than enzyme specificity alone, dictate the metabolic outcomes for these provitamin A carotenoids. This finding broadens the concept of mitochondrial based xanthophyll metabolism and plasma membrane-based carotene metabolism that we previously reported in cell lines (26, 27).

Mouse studies further confirmed the mechanisms observed in cells. In *Bco1*^*−/−*^ mice, α-carotene accumulated due to the inability of BCO2 to compensate, supporting its limited mitochondrial availability. Conversely, β-cryptoxanthin was effectively processed by BCO2, as indicated by the presence of β-10′-apocarotenol in the jejunum and the absence of β-cryptoxanthin accumulation as observed for α-carotene in the liver of the mouse mutant.

We further showed that conversion of α-carotene by BCO1 is regulated by the transcription factor ISX. ISX-deficient mice, characterized by enhanced bioconversion of carotene and chylomicron-mediated vitamin A transport (32), showed elevated α-retinoid levels in the jejunum and liver when compared to WT mice. The total amount of hepatic REs even exceeded the amounts of REs in mice fed with diets supplemented with preformed vitamin A. It remains to be investigated if this massive accumulation of α-retinoids in the liver is associated with any adverse health effects. Additionally, it is not clear whether these α-RE stores persist in mice or undergo further metabolism and excretion from the body.

What we can currently exclude is that hepatic α-retinol is mobilized by RBP4 and transported in the blood since it remained undetectable in the circulation, consistent with the lack of RBP4 binding (15, 16, 29). This lack of blood transport was observed in WT and ISX-deficient mice, although the latter displayed highly elevated levels of α-retinoids when fed with α-carotene. Our study in DKO mice further confirmed that the accumulation of α-retinoid in peripheral tissues, such as the lung, occurs independent of the RBP4-STRA6 axis, confirming the conclusions by others that the distribution of α-retinoids to peripheral tissue depends on chylomicron-mediated transport mechanisms. Interestingly, small amounts of α-retinyl esters appeared in the eyes of ISX-deficient mice, suggesting that altered chylomicron kinetics facilitated some ocular delivery of the compounds. However, ocular α-RE levels remained low, even in DKO mice, where RBP4-mediated delivery of retinoids is disrupted and the eyes depend on retinoids in chylomicrons. This contrasts studies with β-carotene supplementation in DKO mice, which increased ocular retinoid concentration and improved visual performance of the mutant eyes (44).

These findings indicated the presence of additional protective mechanisms within ocular tissues that selectively exclude α-retinoids from participation in the visual cycle. One hypothesis is that retinoid uptake in the retinal pigment epithelium (RPE) discriminates against α-retinoids. However, this is rather unlikely since LRAT, the major driver in this process (35), readily esterified α-retinol. Alternatively, α-retinoids might undergo rapid metabolism or clearance in ocular tissues. Our experiments in LRAT and RPE65 expressing NIH3T3 cells argue against enzymatic conversion of the ε-ionone ring to a β-ionone ring by RPE65, as recently proposed for carotenoids. In this RPE catalyzed reaction, the 3-hydroxy-ε-ionone ring of lutein is converted to a 3-hydroxy-β-ionone ring to yield meso-zeaxanthin (38). Molecular docking experiments showed that the substrate tunnel of RPE65 does not accommodate α-retinyl esters and thus exclude them from the isomerization reaction. This result is consistent with enzyme tests with bovine RPE membranes that revealed that the 5,6 double bond, lacking in α-retinoids, is critical for the isomerization reaction (43). Experiments with NinaB enzyme also revealed selective interaction of with β-ionone ring site of the asymmetric α-carotene. Though RPE65 and NinaB differ in enzymatic catalysis (42), our findings showed that the substrate tunnels of this class of enzymes can distinguish between different types of ionone rings in visual chromophore production.

Taken together, our study underscores that subtle structural differences between the ionone ring sites of carotenoids shape their metabolic routes, tissue targeting, and physiological roles, and uncovers the molecular principles behind this metabolism. The addition of polar hydroxy groups favor transport to mitochondria *via* Aster proteins, whereas a β-ionone ring is critical for enzymatic processing by BCO1 and transport by RBP4. Our findings show that α-retinoids are valuable tracers for studying chylomicron-mediated vitamin A delivery, thereby confirming studies in piglets and gerbils (4, 15). Unlike β-cryptoxanthin, α-carotene bypasses mitochondrial BCO2 and is metabolized exclusively by cytosolic BCO1, resulting in distribution and peripheral accumulation of α-retinoids as esters but exclusion from circulation and the visual cycle. This selective uptake underscores the eye’s reliance on RBP4 for retinoid homeostasis (13, 14) and highlights protective mechanisms that safeguard visual function from noncanonical retinoids. Furthermore, the robust peripheral accumulation of α-retinoids in tissues such as the lung reinforces their utility in dissecting vitamin A distribution pathways in mammals. Future studies need to elucidate the putative physiological functions of these nonconical retinoids as well as the pathways for their degradation.

## Experimental procedures

### Materials

All chemicals, unless otherwise specified, were purchased from Fisher and Sigma-Aldrich. Retinoid standards were purchased from Toronto Research Chemicals. Carotenoids and apocarotenoids were a gift from Dr Adrian Wyss (DSM, Sisslen).

### Plasmid construction

The murine BCO1 plasmid was constructed by subcloning the murine BCO1 gene into the pMAL C5X vector (New England Biolabs) using XmnI and SbfI restriction sites and the sequence was confirmed with Sanger sequencing. The construction of the murine BCO2 plasmid using the same restriction sites into the pMAL C5X vector was previously reported (45).

### BCO1 and BCO2 protein purification and enzyme activity assay

Maltose-binding protein (MBP) fused to murine BCO2 enzyme and BCO1 enzyme was purified as previously described using affinity chromatography (46). In enzyme assays, 100 μg of purified proteins were incubated with the substrate α-carotene (2000 pmol) in detergent decyl maltose neopentyl glycol (DMN) (Anatrace) micelles for 10 min at 35 °C. In BCO1 enzyme activity assays, the reactions were stopped with 100 μl hydroxylamine (2M, pH 6.8) and 100 μl methanol and incubated at room temperature for 10 min. Next, the retinal oxime products and remaining substrate were extracted using 400 μl of acetone and 500 μl of hexane. In BCO2 enzyme assays, substrates and products were extracted using 100 μl of water, 400 μl of acetone, 400 μl of diethyl ether, and 100 μl of petroleum ether. The extracted organic phases were evaporated in a Speedvac microcentrifuge (Eppendorf); and the debris was reconstituted in HPLC solvent.

## NinaB enzyme activity assay

NinaB enzyme activity assay was performed as previously published methods (41). Briefly, *Galleria* NinaB protein was overexpressed in *E. coli* BL21 Codon Plus cells, and the protein was purified using His tag affinity chromatography. The purified protein was used for enzyme activity with α-carotene as described above for BCO1.

### HPLC analyses of carotenoids and apocarotenoids

For the analysis of the composition of carotenoids, apocarotenoids, and retinoids, the compounds were extracted from tissues, cells, and enzyme assays as described in different sections and reconstituted in the appropriate HPLC solvents. Several HPLC systems were used for the analysis of the compounds. The isocratic HPLC system 1 used hexanes: ethyl acetate (90:10 v/v) at a flow rate of 1.4 ml × min^−1^. The gradient HPLC system 2 used hexanes: ethyl acetate (99:1 v/v) as the first solvent from minutes 0 to 15 and then switched to hexanes: ethyl acetate (90:10 v/v) from minute 15 to 35 at a flow rate of 1.4 ml x min^−1^. The gradient HPLC system 3 used hexanes: ethyl acetate (99:1 v/v) as the first solvent from minutes 0 to 10 and then switched to hexanes: ethyl acetate (80:20 v/v) from minute 10 to 35 at a flow rate of 1.4 ml x min^−1^. Separation of compounds on HPLC systems 1 to 3 was achieved on a Restek Pinnacle DB Silica column (200 × 4.6 mm). The gradient HPLC system 4 used hexanes: isopropyl alcohol (99:1 v/v) as the first solvent from minute 0 to 15, then switched to hexanes: isopropyl alcohol (98:2 v/v) from minute 15 to 40 at a flow rate of 1.0 ml x min^−1^. The isocratic HPLC system 5 used hexanes: isopropyl alcohol (95:5 v/v) at a flow rate of 1.0 ml x min^−1^. Separation of compounds was achieved on a CHIRALPAK AD-3 (4.6 × 250 mm) from Daicel (Chiral Technologies). All HPLC analyses were carried out on an Agilent 1260 Infinity Quaternary HPLC system equipped with a pump (G1312C) with an integrated degasser (G1322A), a thermostated column compartment (G1316A), an autosampler (G1329B), a diode-array detector (G1315D), and online analysis software (Chemstation). The HPLC systems were scaled with authentic standards (α-carotene, β-carotene, retinaldehyde, retinol, and β-10′-apocarotenal).

### α-Carotene and β-cryptoxanthin uptake assay

α-Carotene uptake assay was performed as previously described. Briefly, A549 (ATCC) cells were seeded in 100-mm cell culture plates with 2 million cells per plate seeding density using high-glucose DMEM (Gibco) media with 10% fetal bovine serum (Gibco) and 1X anti-antibiotics (Gibco). The cells were grown in a 37 °C and 5% CO_2_ incubator until the cells reached >90% confluency. Then, cells were treated with 10 mM MCD for 2 h, and then cells were treated with 2 mM MCD (Thermo Fisher) and 4 μM α-carotene or β-cryptoxanthin (DSM) for overnight at 37 °C and 5% CO_2_ incubator. The next day, cells were washed with PBS buffer (Gibco) 3 times using 5 ml of PBS, and cells were collected using a scraper. The collected cells were centrifuged for 5 min and fractionated as previously described. The subcellular fractions, plasma membrane, cytosol, and mitochondria were used to extract the carotenoids and analyzed with the HPLC (Agilent, 1260 Infinity) to determine the carotenoid content. The carotenoids were extracted with 100 μl PBS, 100 μl methanol, 400 μl acetone and 200 μl DEE, and 300 μl hexane. The organic phase was separated and vacuum dried, and finally reconstituted in HPLC solvent.

### Mouse strains and husbandry

Animal procedures and experiments were approved by the Case Western Reserve University Institutional Animal Care and Use Committee and conformed to recommendations of both the American Veterinary Medical Association Panel on Euthanasia and the ARVO Statement for the Use of Animals in Ophthalmic and Vision Research. WT (C57BL/6J) mice were purchased from Jackson Laboratory. The generation of *Bco1*^*−/−*^, *Isx*^*−/−*^, and DKO mice has been previously described (17, 32, 47). The different knockout strains were on the same genetic background as WT mice. For breeding, mice were maintained on standard mouse chow in a vivarium at Case Western Reserve University. For the carotenoid feeding studies, mice were subjected to feeding a vitamin A-deficient purified diet (AIN93 G formulation, Research Diets Inc.) for 1 week. Then, they were fed with the same diet supplemented with α-carotene (50 mg/kg) or β-cryptoxanthin (50 mg/kg) for 10 days. At the end of the feeding period, mice were anesthetized by intraperitoneal injection of a mixture containing ketamine 20 mg, Xylazine 7.5 mg, and sterile water or saline, with a dose of 0.2 ml per 25 g of mouse. Blood was drawn directly from the heart *via* cardiac puncture. Mice were then perfused with 20 ml PBS and killed by cervical dislocation. Tissues were removed for analyses and/or snap-frozen in liquid nitrogen and stored at −80 °C until use.

### Carotenoid and retinoid extractions from mouse tissues

10 to 20 mg of mouse tissue sample were analyzed. In α-carotene feeding experiments, tissues were extracted with 100 μl PBS, 100 μl methanol, 400 μl acetone, and 500 μl hexanes. The organic layers were separated, vacuum dried, and reconstituted in HPLC solvent. For ocular extractions, one eye was used. Additionally, extraction was performed in the presence of hydroxylamine (2M, pH 6.8) to convert retinaldehydes into the corresponding oximes as previously described (44). β-Cryptoxanthin and its metabolites were extracted using 100 μl PBS, 100 μl methanol, 400 μl acetone, 200 μl diethyl ether, and 500 μl petroleum ether. The organic layer was separated, vacuum dried, and reconstituted in HPLC solvent.

### Cell based LRAT enzyme assays

Retinoids from α-carotene-supplemented mouse liver (10 mg) was extracted with 200 μl PBS, 200 μl methanol, 400 μl acetone, and 500 μl hexane. The extracted organic phase was vacuum-dried and saponified. Briefly, the dried retinoid extract was redissolved in saturated KOH ethanolic solution and incubated at 42 °C, and reextracted using hexane. Both liver extraction and saponified liver extractions were run on HPLC system 4 to confirm the saponification of retinyl esters to retinol.

NIH3T3 cell line expressing LRAT (31) was seeded in 100 mm plates and grew to >90% confluent. Then cells were serum-starved overnight. Next cells were treated with retinoids from saponified liver extract dissolved in DMSO (0.1% v/v final concentration) for overnight in the serum-free media. Then cells were washed with PBS buffer three times using 15 ml PBS buffer per plate. Then, cells were collected by scraping, harvested by centrifugation, and stored at −80 °C. Finally, the cells were extracted using 100 μl PBS, 100 μl methanol, 400 μl acetone, and 500 μl hexane. The organic layer with extracted retinoids was vacuum dried and reconstituted in HPLC solvent.

### Western blot

Serum (1 μl) from mice was subjected to SDS-PAGE (12%), and proteins were transferred onto membranes as previously described (32). Serum albumin was used as the loading control, and Ponceau S staining was used for serum albumin staining. The primary antibody RBP4 (Dako, Denmark) was used at 1:500 dilution and was incubated overnight at 4 °C. The secondary rabbit antibody was used in 1:5000 dilution as previously validated (32).

### Structure alignment and docking

The RPE65 crystal structure (PDB ID 4RSC) is used to dock retinyl acetate into the substrate binding cavity. The ligands retinyl acetate and α-retinyl acetate were docked using the ProteniX server. The protein structure and dock ligands were aligned, and figures were created using PyMOL software.

### Statistical analysis

Data shown are the mean ± SD. Analysis was performed using an unpaired two-tailed *t* test using Graphpad Prism 10.2 software, and results were considered significant at ∗*p* < 0.05, ∗∗*p* < 0.005, ∗∗∗*p* < 0.0001.

## Data availability

All data are contained within the article.

## Supporting information

This article contains supporting information.

## Conflict of interest

The authors declare that they have no conflicts of interest with the content of this article.
