# Supplementary material for: Structural bias in vitamin A metabolism: Why α-retinoids miss the eye
Source: J Biol Chem. 2025 Sep 11;301(10):110713. doi: 10.1016/j.jbc.2025.110713 (PMC12550161; doi:10.1016/j.jbc.2025.110713)
Supplement: Supporting_information [file mmc1.docx]

**Supporting Information**

**Structural Bias in Vitamin A Metabolism: Why α-Retinoids Miss the Eye**

Sepalika Bandara^1^, Aicha Saadane^1^, Pranesh Ravichandran^1,2^, Ramkumar Srinivasagan^1,3^, and Johannes von Lintig^1*^

^1^Department of Pharmacology, School of Medicine, Case Western Reserve University, Cleveland, OH, 44106, USA.

^2^ Department of Surgery, University of Illinois College of Medicine at Peoria, Peoria, IL, 61605, USA

^3^ Disease Biology Laboratory, Baush & Lomb Incorporated, Irvine, CA, USA

**Corresponding author: *** [johannes.vonlintig@case.edu](mailto:johannes.vonlintig@case.edu)

**Supplementary Figures**


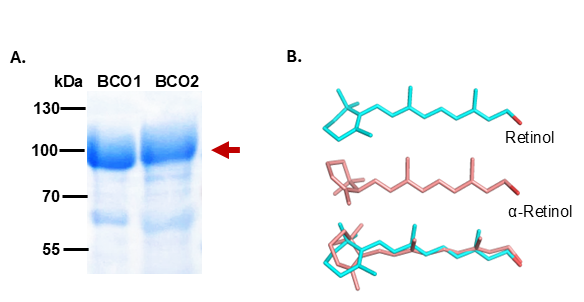


**Figure S1.** A. SDS-PAGE with affinity column purified mouse BCO1 and BCO2 MBP fusion proteins. B. 3D structures of all-trans-retinol (cyan), all-trans-α-retinol (pink), and the overlayed structures.

**
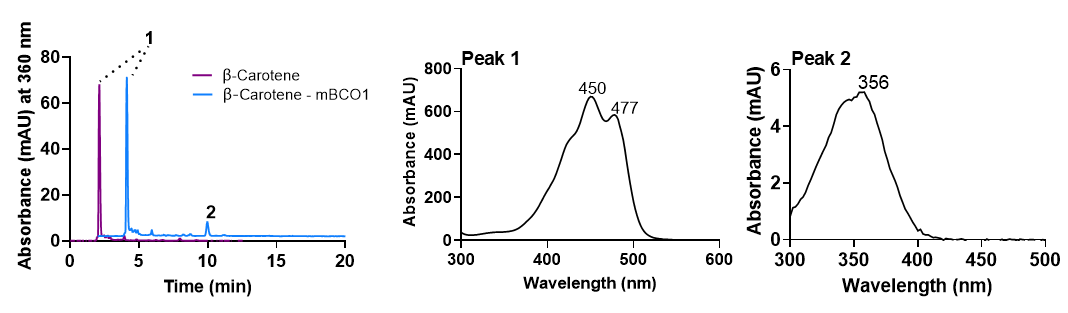
**

**Figure S2.** β-Carotene conversion by recombinant BCO1. **A**. HPLC traces at 360 nm of a β-carotene standard (purple trace) and β-carotene incubated in the presence of mouse BCO1 enzyme (blue trace). **B**. Spectral characteristics of peak 1, β-carotene and peak, 2, all-*trans*-retinal oxime (syn).

**
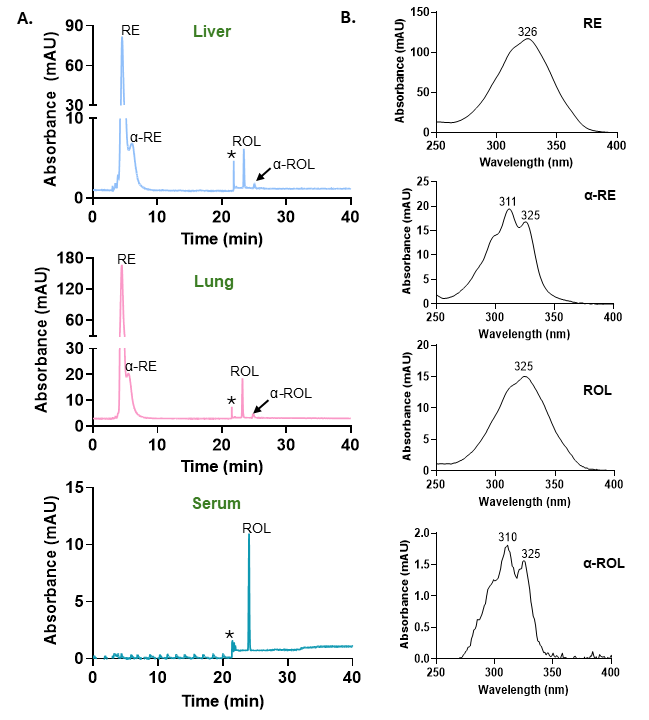
**

**Figure S3. α-Carotene metabolism of wild type mice. A.** HPLC chromatogram of tissue extracts of wild type mice fed with α-carotene. The retentions times of α-retinol (α-ROL) and α-retinyl esters (α-REs) and retinol (ROL) and retinyl esters (RE) is indicated. The asterisks (*) mark a peak that results from the change in solvent composition during the gradient elution. **B.** UV-visible spectra of RE, α-RE, ROL and α-ROL. Separation was achieved on HPLC system 4.

**
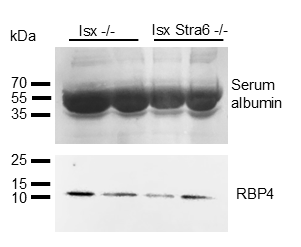
**

**Figure S4.** Western blot for RBP in mice supplemented with α-carotene. Ponceau-S staining for serum albumin was used as loading control.

**
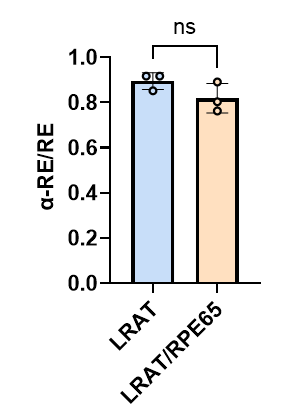
**

**Figure S5.** NIH3T3 cells expressing LRAT (blue) alone and LRAT and RPE65 (orange) together were incubated with a mixture of retinol and α-retinol extracted from mouse liver. After 16 hours, retinoids were extracted and separated by HPLC system 5. Retinyl esters were quantified, and their ration was blotted in the graph. We observed no significant difference between cell lines, indicating that RPE65 does not contribute to the conversion of α-retinyl ester (α-RE) to retinyl ester (RE). Note that 11-cis-retinol produced by RPE65 will be esterified by LRAT in this test system. n.s., no significant difference.
